# Effects of Exercise on Aerobic Capacity in People with Chronic Obstructive Pulmonary Disease: A Systematic Review and Meta-Analysis of Randomized Controlled Trials

**DOI:** 10.3390/healthcare14142165

**Published:** 2026-07-17

**Authors:** Lin Hu, Yifan Zhang, Xiang Li, Yin Liang, Yuanyuan Lv, Laikang Yu

**Affiliations:** 1 Beijing Key Laboratory of Sports Performance and Skill Assessment, Beijing Sport University, Beijing 100084, China; hu1533119449@163.com; 2 Department of Strength and Conditioning Assessment and Monitoring, Beijing Sport University, Beijing 100084, China; 18738314013@163.com (Y.Z.); jinaxun0889@yeah.net (X.L.); 18076397318@163.com (Y.L.); 3 China Institute of Sport and Health Science, Beijing Sport University, Beijing 100084, China; sunflowerlyy@bsu.edu.cn

**Keywords:** exercise, chronic obstructive pulmonary disease, aerobic capacity, 6 min walk distance

## Abstract

**Background/Objectives**: This study aimed to evaluate the impact of exercise on aerobic capacity in individuals with chronic obstructive pulmonary disease (COPD), and to explore potential exercise-related characteristics associated with greater improvements in aerobic capacity. **Methods**: Randomized controlled trials (RCTs) were systematically retrieved from Web of Science, Embase, Scopus, PubMed, and Cochrane Library, covering all records up to 13 May 2025. Studies were included if they were RCTs involving COPD patients and reported aerobic capacity outcomes. Methodological quality was evaluated using the Cochrane Risk of Bias tool, and effect sizes were synthesized using weighted mean differences (WMD) with 95% confidence intervals (CIs). **Results**: Thirty-seven studies were included, involving 1124 participants in exercise groups and 1089 in control groups. Exercise significantly improved aerobic capacity (WMD, 49.82; 95% CI: 38.54 to 61.10; *p* < 0.00001). Subgroup analyses revealed that certain exercise characteristics, including mixed exercise (WMD, 53.70; 95% CI: 41.58 to 65.83; *p* < 0.00001), sessions ≥ 60 min (WMD, 51.15; 95% CI: 37.53 to 64.76; *p* < 0.00001), ≤3 times weekly (WMD, 62.09; 95% CI: 47.49 to 76.68; *p* < 0.00001), total weekly volume ≤ 180 min (WMD, 59.08; 95% CI: 41.52 to 76.64; *p* < 0.00001), and supervised training (WMD, 56.25; 95% CI: 36.64 to 75.86; *p* < 0.00001), may be associated with larger improvements in aerobic capacity. **Conclusions**: Exercise is effective in improving aerobic capacity in COPD patients. Observed differences across exercise characteristics should be considered as exploratory and hypothesis-generating rather than definitive evidence of optimal exercise prescription. Future research should focus on investigating the long-term effects of exercise training on clinical outcomes (e.g., hospital readmissions, mortality) and exploring the efficacy of technology-assisted remote exercise programs to improve access to pulmonary rehabilitation services.

## 1. Introduction

Chronic obstructive pulmonary disease (COPD) is a progressive respiratory disorder characterized by persistent airflow limitation and chronic respiratory symptoms. Although COPD is both preventable and treatable, its associated mortality and morbidity continue to pose substantial global public health challenges, with the overall disease burden steadily increasing. It is currently the third leading cause of death worldwide [1], and the number of affected individuals is projected to reach 600 million by 2050 [2].

COPD is commonly accompanied by comorbidities such as cardiovascular disease, respiratory failure, and lung cancer. These conditions, together with the underlying pathophysiology of COPD, often contribute to a progressive decline in aerobic capacity—a key determinant of health-related quality of life (HRQOL). In this study, aerobic capacity primarily refers to functional exercise capacity assessed using field-based tests, predominantly the 6 min walk distance (6MWD), rather than directly measured peak oxygen uptake (VO_2_peak). As aerobic capacity deteriorates, HRQOL correspondingly diminishes. Therefore, strategies aimed at improving aerobic capacity may represent an effective approach to enhancing overall well-being in individuals with COPD.

Current COPD management includes both pharmacological and non-pharmacological interventions. Standard treatment, such as bronchodilators, inhaled corticosteroids, and theophylline, can alleviate symptoms but may also produce adverse effects, including tachycardia, dizziness, headache, dysuria, nausea, and vomiting. Although pharmacological therapies have been shown to reduce dyspnea in COPD patients [3], previous research [4] together with the 2020 GOLD Guidelines [5] have consistently highlighted the central role of pulmonary rehabilitation (PR) in disease management. Exercise, as a core component of PR, is increasingly recognized for its capacity to improve aerobic capacity and consequently enhance HRQOL.

Although numerous studies have investigated the effects of exercise on aerobic capacity in individuals with COPD, the findings remain inconsistent. Abdelbasset et al. [6] reported significant improvements in aerobic capacity following a 12-week endurance training program in obese older adults with COPD. Similarly, Kantatong et al. [7] demonstrated that a 24-week Tai Chi Qigong intervention enhanced aerobic capacity and HRQOL. Collectively, these results suggest that exercise may serve as an effective intervention for enhancing functional exercise performance in this population. However, contrasting evidence exists; for instance, Moy et al. [8] observed no significant changes in aerobic capacity following 24 weeks of Tai Chi training. Such discrepancies may reflect differences in exercise type, intensity, intervention duration, and the level of professional supervision.

A previous meta-analysis has investigated the combined effects of aerobic exercise and breathing training on stable COPD and reported improvements in aerobic capacity and HRQOL [9]. However, methodological issues limited the interpretability of its findings: control groups also received exercise interventions, whereas experimental groups underwent non-exercise breathing components, which complicates the isolation of exercise-specific effects. In addition, although PR has been shown to improve lung function and aerobic capacity [10], PR programs comprise multiple therapeutic components beyond exercise. The study did not clearly distinguish between PR-based interventions and exercise-only interventions, nor did it offer explicit recommendations regarding optimal exercise prescription. Another meta-analysis examining aerobic exercise in COPD patients was limited by its focus on aerobic training alone, lacking a comprehensive assessment of diverse exercise modalities (e.g., frequency, intensity, session duration, weekly time, and supervision status) and providing no guidance on selecting the most effective exercise program [11]. Additionally, the use of heterogeneous outcome measures and considerable variability in exercise interventions has hindered meaningful comparisons and synthesis of evidence.

In recent years, exercise interventions for COPD have become increasingly diverse in format, duration, intensity, and supervision, providing broader rehabilitation options but also emphasizing the need for updated and systematic evaluation. Therefore, this systematic review and meta-analysis aimed to evaluate whether existing evidence supports the efficacy of exercise in improving aerobic capacity and to determine which exercise-related characteristics are associated with the most favorable aerobic outcomes.

## 2. Materials and Methods

### 2.1. Design

This systematic review and meta-analysis were conducted in accordance with the Cochrane Handbook for Systematic Reviews of Interventions [12] and the Preferred Reporting Items for Systematic Reviews and Meta-Analyses (PRISMA) guideline [13]. The study protocol was prospectively registered in PROSPERO (CRD420251063335).

### 2.2. Search Strategy

A comprehensive literature search was performed in Web of Science, Embase, Scopus, PubMed, and Cochrane Library from database inception to 13 May 2025. Studies examining exercise interventions and aerobic capacity in individuals with COPD were identified using combinations of MeSH terms and free-text keywords, including “exercise”, “COPD”, and “aerobic capacity” (Table S1). To minimize the likelihood of omitting relevant studies, we additionally hand-searched the reference lists of all included articles as well as related review and prior meta-analyses.

Study identification and screening were completed independently by two authors (L.H. and Y.Z.) following a standardized protocol. Disagreements were resolved through discussion, and when consensus was not reached, a third author (LY) served as arbitrator. Final inclusion decisions required agreement from all three authors.

### 2.3. Eligibility Criteria

Studies were considered eligible if they met the following criteria: (1) randomized controlled trial (RCT) design; (2) inclusion of both an intervention group and a control group; (3) participants diagnosed with COPD; (4) reporting outcome indicators related to aerobic capacity. Studies were excluded if they were non-English publications, animal experiments, review articles, or conference abstracts.

### 2.4. Data Extraction

Data were extracted independently by two authors (L.H. and Y.Z.) using a pre-designed standardized data extraction form. Extracted information included: (a) study characteristics (first author, publication year, sample size); (b) intervention details (type, intervention duration, frequency, session duration, and supervision format); (c) participant characteristics (age and body mass index [BMI]); and (d) outcome data, specifically mean values and standard deviations (SDs) for changes in aerobic capacity from baseline to post-intervention. Any discrepancies were resolved through discussion, with adjudication by a third author (L.Y.) when necessary.

In cases of missing or incomplete data, attempts were made to contact study authors for clarification. When necessary, missing statistical data were calculated from available information such as standard errors, confidence intervals, or *p*-values according to Cochrane Handbook guidelines.

### 2.5. Methodological Quality Assessment

The methodological quality of the included studies was evaluated using the Cochrane Risk of Bias tool (RoB 1), which examines 7 domains: random sequence generation (selection bias), allocation concealment (selection bias), blinding of participants and personnel (performance bias), blinding of outcome assessment (detection bias), incomplete outcome data (attrition bias), selective reporting (reporting bias), and other biases. RoB 1 was adopted rather than RoB 2 because a substantial proportion of the included RCTs were conducted and reported prior to the introduction of RoB 2, and several studies did not provide sufficient methodological detail required for the domain-level signaling questions of RoB 2, particularly regarding deviations from intended interventions and trial protocol information. Therefore, RoB 1 was considered more appropriate and consistent for ensuring uniform assessment across all included studies.

Each domain was classified as “low risk”, “unclear risk”, or “high risk” based on the Cochrane Handbook predefined signaling questions. The assessment was independently performed by two authors (L.H. and Y.Z.), and any discrepancies were resolved through discussion with a third author (L.Y.) until consensus was reached.

### 2.6. Certainty Assessment

The certainty of evidence across outcomes was evaluated using the Grading of Recommendations Assessment, Development and Evaluation (GRADE) framework, with evidence quality classified as high, moderate, low, or very low. The certainty of evidence was downgraded based on limitations in risk of bias, inconsistency, indirectness, imprecision, and potential publication bias, and upgraded when large effect sizes or consistent and robust findings across studies were observed. The GRADE assessment was conducted by two independent authors (L.H. and Y.Z.).

### 2.7. Statistical Analysis

For each study, mean values and SDs of the 6MWD were extracted directly from the original articles whenever available. When change scores and their corresponding SDs were not directly reported, they were calculated from pre- and post-intervention values using the formulas recommended by Higgins et al. [12]. Pooled effect sizes were synthesized using either fixed-effects or random-effects models according to the level of between-study heterogeneity. Pooled effect sizes were synthesized using either fixed-effects or random-effects models according to the level of between-study heterogeneity. Specifically, a fixed-effects model was applied when heterogeneity was low (I^2^ < 50%), assuming a common underlying true effect across studies, whereas a random-effects model was used when heterogeneity was substantial (I^2^ ≥ 50%), assuming that true effects may vary across studies. All pooled estimates were expressed as weighted mean difference (WMD) with 95% confidence intervals (CIs). In the presence of substantial heterogeneity (I^2^ > 50%), subgroup analyses, meta-regression, and sensitivity analyses were performed to explore potential sources of variability and to test robustness of the results [14]. Publication bias was evaluated through visual inspection of funnel plots and quantitatively assessed using Egger’s test.

Meta-regression analyses were performed when at least 10 studies were available for a given outcome, in line with recommended methodological guidance for ensuring stable estimates and reducing the risk of overfitting. The variables entered into the meta-regression models were selected a priori based on clinical relevance and previously reported exercise prescription parameters in COPD populations, including intervention type, frequency, session duration, weekly time, and supervision status.

Subgroup analyses explored whether exercise effects differed according to: (1) intervention type (aerobic exercise vs. mixed exercise), (2) session duration (<60 min per session vs. ≥60 min per session), (3) frequency (≤3 times per week vs. >3 times per week), (4) weekly time (≤180 min per week vs. >180 min per week), and (5) supervision level during training (supervised vs. unsupervised).

Meta-analyses were conducted in RevMan 5.4, whereas meta-regression, sensitivity analyses, and funnel plots for publication bias were performed in Stata 19. A two-sided *p*-value < 0.05 was considered statistically significant.

## 3. Results

### 3.1. Study Selection

**Figure 1:**
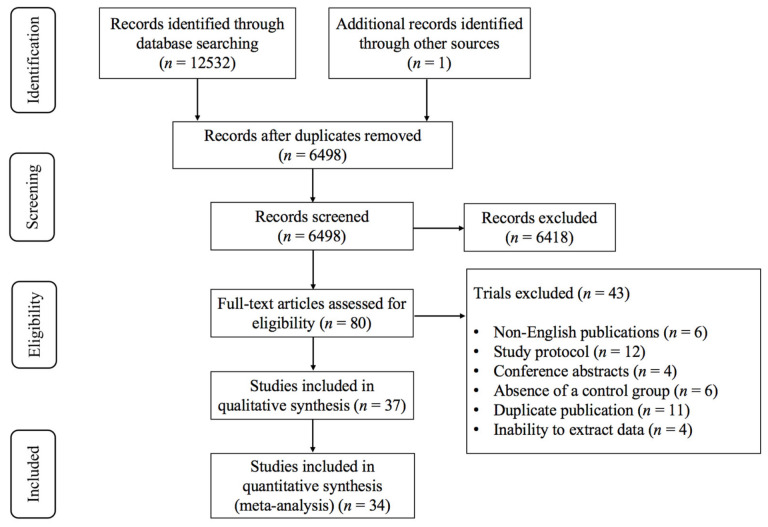
PRISMA flowchart of study selection.

As shown in Figure 1, a total of 12,532 records were identified through database searches, and one additional record was retrieved from other sources. After removing duplicate studies, 6498 studies remained. Following title and abstract screening, 6418 studies were excluded for not meeting eligibility criteria. Full texts of the remaining 80 studies were assessed, and 43 studies were excluded for the following reasons: (1) non-English publications (*n* = 6); (2) study protocols (*n* = 12); (3) conference abstracts (*n* = 4); (4) absence of a control group (*n* = 6); (5) inability to extract data (*n* = 4); and (6) duplicate publications (*n* = 11). Ultimately, 37 studies [6,7,8,15,16,17,18,19,20,21,22,23,24,25,26,27,28,29,30,31,32,33,34,35,36,37,38,39,40,41,42,43,44,45,46,47,48] met the eligibility criteria and 34 studies [6,7,8,15,16,17,18,19,20,21,22,23,24,25,26,27,28,29,30,32,33,34,35,38,39,40,41,42,43,44,45,46,47,48] were selected for meta-analysis.

### 3.2. Study Characteristics

Key characteristics of the included studies are presented in Table S2. Across 37 studies, 1124 participants assigned to 41 exercise groups and 1089 participants to 37 control groups. Four studies were three-armed trials [22,30,35,46]. Gender distribution was reported in 33 studies, while 4 studies [16,22,31,36] did not provide sex-specific data; 4 studies [41,43,44,45] included only male patients. All included studies assessed aerobic capacity: 34 studies [6,7,8,15,16,17,18,19,20,21,22,23,24,25,26,27,28,29,30,32,33,34,35,38,39,40,41,42,43,44,45,46,47,48] used the 6MWD, whereas 3 studies [31,36,37] used alternative indicators (peak oxygen uptake, 2 min walk distance, or endurance shuttle walk). Only studies reporting 6MWD outcomes were included in the quantitative synthesis.

Of the 37 studies, 23 studies incorporated supervised training [6,8,15,16,17,18,20,22,23,24,25,27,29,30,31,32,35,36,37,39,41,43,47], 9 studies [19,21,26,34,38,42,44,45,48] employed unsupervised training, and 5 studies [7,28,33,40,46] included both supervised and unsupervised components. Intervention types included whole-body vibration training, aerobic exercise, resistance exercise, Tai Chi, yoga, Qigong, and aquatic training. Intervention duration ranged from 1 to 48 weeks, with session durations ranging from 8 to 110 min and weekly frequencies from 2 to 14 sessions. Total weekly training volume—calculated from session duration and frequency—ranged from 120 to 540 min.

### 3.3. Meta-Analysis

**Figure 2:**
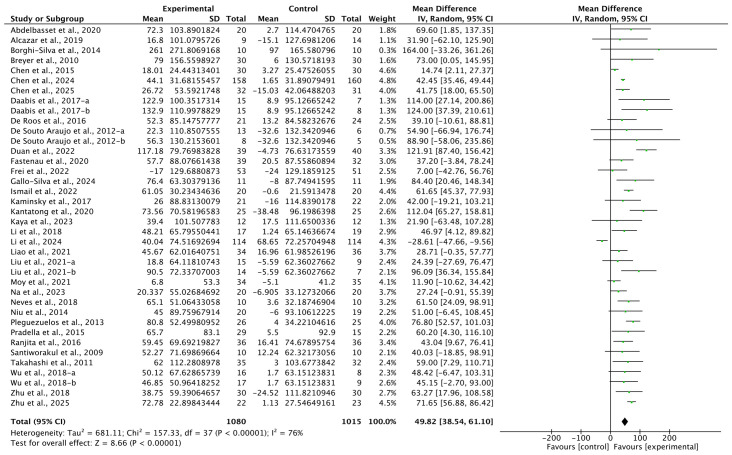
Meta-analysis results of the effect of exercise on aerobic capacity in COPD patients [6,7,8,15,16,17,18,19,20,21,22,23,24,25,26,27,28,29,30,32,33,34,35,38,39,40,41,42,43,44,45,46,47,48].

Across 34 studies (38 effect sizes), including four three-armed trials contributing multiple effect sizes, exercise significantly improved aerobic capacity in COPD patients (WMD, 49.82; 95% CI: 38.54 to 61.10; *p* < 0.00001; I^2^ = 76%, Figure 2).

### 3.4. Meta-Regression

As shown in Table S3, meta-regression analyses revealed no significant associations between changes in 6MWD and session duration (*p* = 0.450), weekly time (*p* = 0.262), intervention type (*p* = 0.990), or supervision status (*p* = 0.266). In contrast, exercise frequency was significantly associated with changes in 6MWD (*p* = 0.003).

### 3.5. Subgroup Analyses

**Figure 3:**
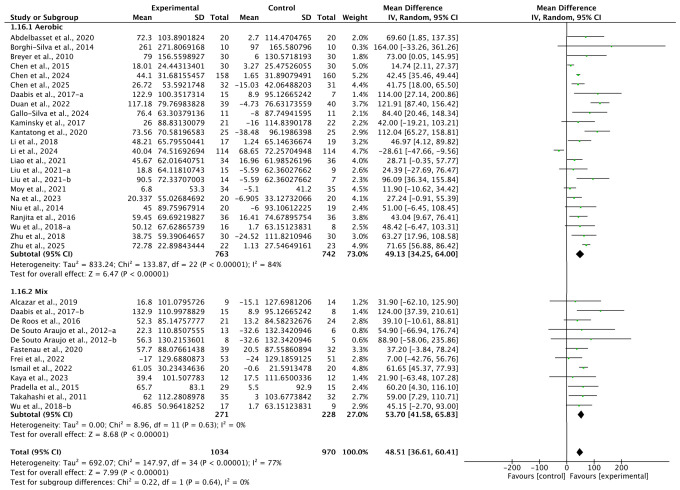
Meta-analysis results of the effect of types of intervention on aerobic capacity in COPD patients [6,7,8,15,16,17,18,19,20,21,22,23,24,25,26,27,28,29,30,32,33,34,35,38,39,40,41,42,43,45,46,47,48].

Twenty-two studies implemented aerobic exercise, and 11 studies used mixed exercise programs. Aerobic exercise (WMD, 49.13; 95% CI: 34.25 to 64.00; *p* < 0.00001; I^2^ = 84%) and mixed exercise (WMD, 53.70; 95% CI: 41.58 to 65.83; *p* < 0.00001; I^2^ = 0%, Figure 3) significantly improved aerobic capacity in COPD patients. Mixed exercise was associated with a numerically larger pooled effect size compared with aerobic exercise alone.

**Figure 4:**
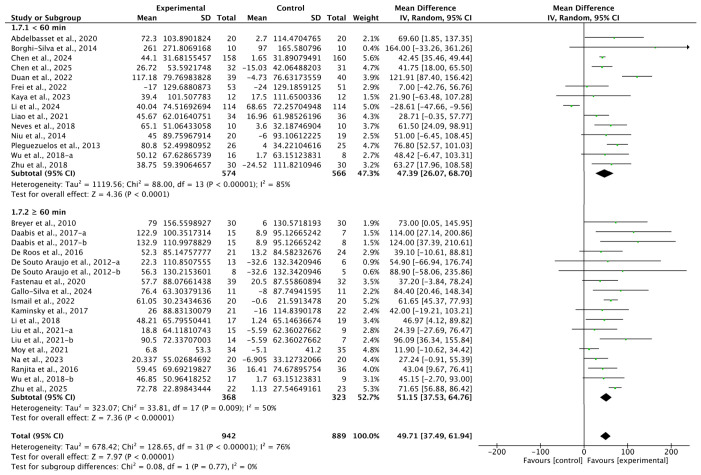
Meta-analysis results of the effect of duration of intervention per session on aerobic capacity in COPD patients [6,8,16,17,18,19,20,22,23,24,25,26,27,28,30,32,33,34,35,38,39,40,41,43,46,47,48].

Fourteen studies involved sessions < 60 min, and 15 studies involved sessions ≥ 60 min. Sessions < 60 min (WMD, 47.39; 95% CI: 26.07 to 68.70; *p* < 0.0001, I^2^ = 85%) and sessions ≥ 60 min (WMD, 51.15; 95% CI: 37.53 to 64.76; *p* < 0.00001; I^2^ = 50%, Figure 4) significantly improved aerobic capacity in COPD patients. Sessions ≥ 60 min were associated with a slightly larger pooled effect size than shorter sessions.

**Figure 5:**
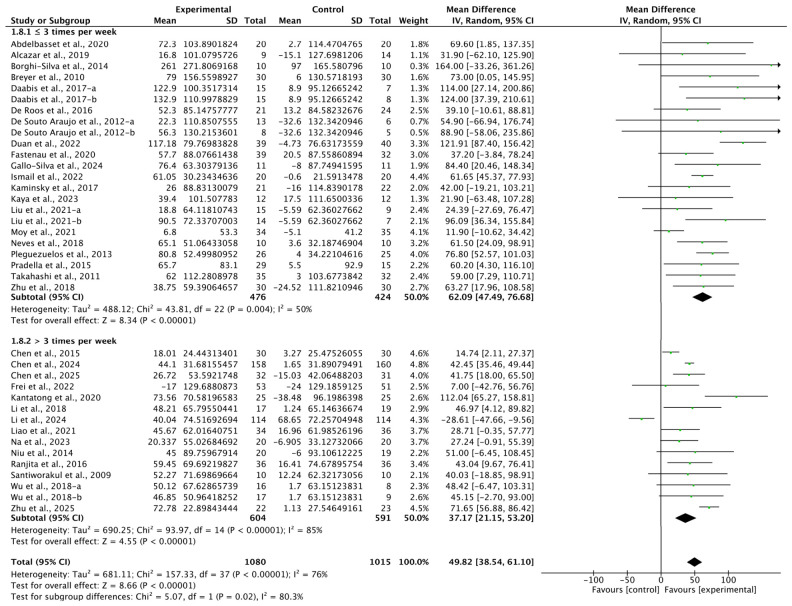
Meta-analysis results of the effect of frequency of intervention on aerobic capacity in COPD patients [6,7,8,15,16,17,18,19,20,21,22,23,24,25,26,27,28,29,30,32,33,34,35,38,39,40,41,42,43,44,45,46,47,48].

Twenty studies implemented frequencies ≤ 3 times per week, and 14 studies implemented frequencies > 3 times per week. Frequencies ≤ 3 times per week (WMD, 62.09; 95% CI: 47.49 to 76.68; *p* < 0.00001; I^2^ = 50%) and frequencies > 3 sessions per week (WMD, 37.17; 95% CI: 21.15 to 53.20; *p* < 0.00001; I^2^ = 85%, Figure 5) significantly improved aerobic capacity in COPD patients. Lower-frequency interventions (≤3 times per week) were associated with a larger pooled effect size compared with higher-frequency interventions.

**Figure 6:**
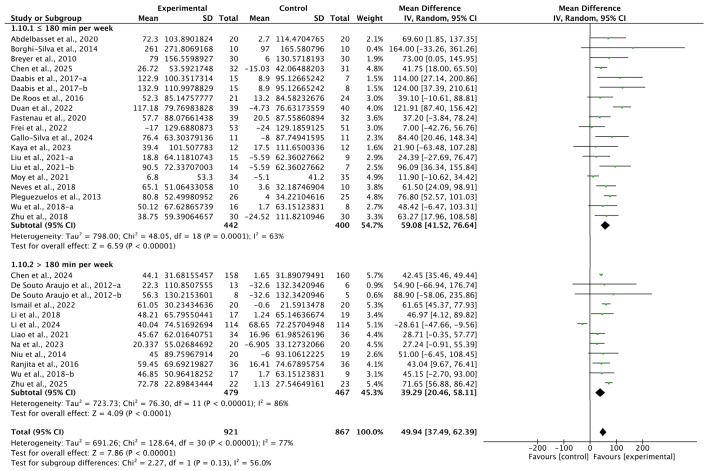
Meta-analysis results of the effect of duration of intervention per week on aerobic capacity in COPD patients [6,8,16,17,18,19,20,22,23,24,25,26,27,28,30,32,33,34,35,38,39,40,41,43,46,47,48].

Seventeen studies reported weekly time ≤ 180 min per week, and 11 studies reported weekly time > 180 min per week. Weekly time ≤ 180 min per week (WMD, 59.08; 95% CI: 41.52 to 76.64; *p* < 0.00001; I^2^ = 63%) and weekly time > 180 min per week (WMD, 39.29; 95% CI: 20.46 to 58.11; *p* < 0.0001; I^2^ = 86%, Figure 6) significantly improved aerobic capacity in COPD patients. Programs with ≤180 min per week were associated with a larger pooled effect size.

**Figure 7:**
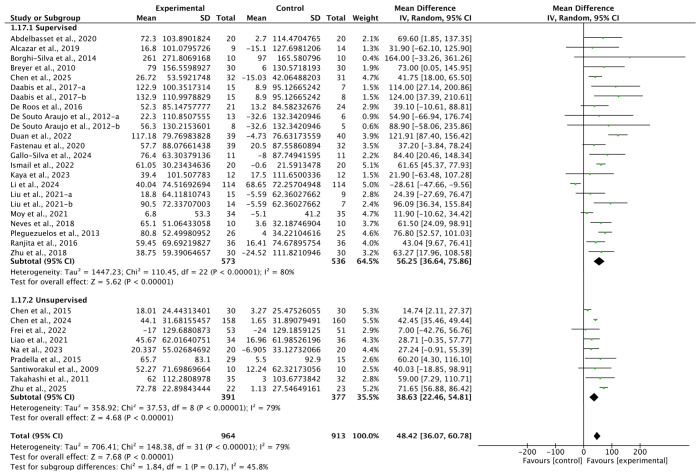
Meta-analysis results of the effect of supervision status on aerobic capacity in COPD patients [6,8,15,16,17,18,19,20,21,22,23,24,25,26,27,29,30,32,34,35,38,39,41,42,43,44,45,47,48].

Twenty studies included professional supervision, while 9 studies were unsupervised. Supervised interventions (WMD, 56.25; 95% CI: 36.64 to 75.86; *p* < 0.00001; I^2^ = 80%) and unsupervised interventions (WMD, 38.63; 95% CI: 22.46 to 54.81; *p* < 0.00001; I^2^ = 79%, Figure 7) significantly improved aerobic capacity in COPD patients. Supervised interventions were associated with a larger pooled effect size compared with unsupervised interventions.

### 3.6. Risk of Bias

Risk of bias was evaluated using the Cochrane Risk of Bias tool across 7 domains. Studies were classified as high-, moderate-, or low-quality according to predefined criteria: (1) either random sequence generation or allocation concealment was judged to be at high risk of bias (low quality); (2) both domains were assessed as low risk and all other domains were rated as either low or unclear risk (high quality); (3) not meeting the above criteria (moderate quality). Among the 37 included studies, 7 were rated as high quality, 29 as moderate quality, and 1 as low quality (Figure S1).

### 3.7. Publication Bias

Funnel plots indicated no visible asymmetry (Figure S2). Egger’s test confirmed the absence of significant publication bias for 6MWD (*p* = 0.156, Table S4).

### 3.8. Sensitivity Analysis

Sensitivity analyses indicated that the pooled effect estimates were robust, with no individual study disproportionately influencing the overall results (Figure S3).

### 3.9. GRADE Summary

The certainty of the evidence was evaluated using the GRADE framework. Following this assessment, the overall certainty of evidence was rated as low, mainly attributable to concerns regarding performance bias and substantial heterogeneity (Table S5).

## 4. Discussion

### 4.1. Main Findings

Our results demonstrated that exercise significantly improved aerobic capacity. Subgroup analyses further revealed that mixed exercise programs, sessions lasting ≥ 60 min, frequencies ≤ 3 times per week, weekly time ≤ 180 min, and professionally supervised interventions were associated with larger pooled effect estimates in aerobic capacity.

### 4.2. Effects of Exercise on Aerobic Capacity in COPD Patients

Consistent with prior studies [49,50], the present study confirms that exercise training yields substantial benefits for aerobic capacity in patients with COPD, aligning with the findings of a previous meta-analysis by Lu et al. [11]. To contextualize these outcomes, it is critical to first consider the pathophysiological mechanisms underlying impaired aerobic capacity in this population. Aerobic capacity in COPD is primarily compromised by persistent airflow limitation, which impairs alveolar gas exchange and reduces oxygen uptake and utilization during physical activity. This deficit in oxygen delivery leads to early onset of exercise fatigue, prompting patients to adopt a sedentary lifestyle; this in turn exacerbates deconditioning of the cardiovascular and musculoskeletal systems, creating a self-perpetuating cycle of declining aerobic capacity [51,52].

The mechanisms by which exercise enhances aerobic capacity in COPD can be elucidated from several physiological perspectives. First, exercise may enhance pulmonary function by improving ventilatory mechanics and gas exchange efficiency [53,54,55], thereby augmenting oxygen transport to working muscles. However, conflicting evidence exists regarding the effects of exercise on lung function (e.g., forced expiratory volume in one second) in COPD [11,56], likely due to heterogeneity in patient severity, baseline functional status, and exercise protocol design. Second, exercise strengthens the respiratory muscle (e.g., diaphragm, intercostal muscles) [57], which improves ventilatory efficiency and reduces the work of breathing during physical activity, thereby delaying the onset of dyspnea and enhancing exercise tolerance [9]. Third, exercise improves skeletal muscle function by increasing mitochondrial density, oxidative enzyme activity, and capillary perfusion [58], which enhances muscle oxygen utilization and reduces fatigue during exercise. Given that peripheral muscle dysfunction is a key contributor to exercise intolerance in COPD, this mechanism likely plays a central role in the observed improvements in aerobic capacity. Finally, exercise exerts anti-inflammatory effects by reducing systemic levels of pro-inflammatory cytokines (e.g., tumor necrosis factor-α, interleukin-6) [59], which may mitigate cardiopulmonary inflammation and improve vascular function, further supporting aerobic performance.

Importantly, when integrating our findings with previous meta-analyses, the present results are broadly consistent in demonstrating that exercise improves aerobic capacity in COPD patients, but differences in magnitude and moderator effects may be explained by variations in intervention classification, outcome definitions, and inclusion criteria across studies.

The 6MWD is the most widely used clinical tool for assessing aerobic capacity and functional exercise tolerance in COPD patients [60], as it reflects the integrated physiological response of the cardiovascular, respiratory, and musculoskeletal systems during submaximal exercise. The observed improvement of 46.66 m in 6MWD in the present study is clinically meaningful, as a decline of 30 m or more in 6MWD has been associated with increased risk of death (but not hospitalization due to exacerbation) in patients with COPD, and this threshold is considered a clinically important minimally important difference [61]. Overall, these findings reinforce the role of exercise as a cornerstone of non-pharmacological management for COPD patients.

### 4.3. Effects of Various Exercise Moderators on Aerobic Capacity in COPD Patients

Subgroup analyses showed that both aerobic and mixed exercise interventions were associated with significant improvements in aerobic capacity. Notably, mixed exercise was associated with a numerically larger pooled effect size compared with aerobic exercise alone. This observation is consistent with the fact that all mixed exercise programs included in the present study incorporated aerobic training as a core component, which is known to enhance cardiovascular fitness [11]. The superior efficacy of mixed exercise interventions can be attributed to the synergistic effects of combining aerobic exercise with resistance exercise. Previous studies have demonstrated that combined aerobic and resistance exercise programs are more effective than aerobic exercise alone for improving exercise capacity in COPD [62,63], as resistance exercise enhances skeletal muscle strength and mass, which reduces the physiological burden of physical activity and delays the onset of fatigue [54]. Additionally, resistance exercise has been shown to reduce exertional dyspnea in COPD patients by improving the efficiency of the respiratory pump and reducing the work of breathing during exercise [64,65]. Furthermore, mixed exercise interventions may reduce cardiovascular load during physical activity and increase muscle oxygenation, thereby optimizing aerobic capacity [66].

The subgroup analysis indicated that sessions of ≥60 min were associated with a slightly larger pooled effect estimate compared with shorter sessions, with substantially lower heterogeneity in the longer session subgroup. This pattern may suggest a potential relationship between session duration and magnitude of improvement within the observed data range. Previous studies have similarly reported that longer exercise sessions are associated with greater improvements in quality of life in COPD [67,68]. However, Beauchamp et al. [67] caution that the effects of session duration may be modified by patient-specific factors (e.g., gender, disease severity). It is important to note that session duration must be balanced against patient tolerance, as excessively long sessions may increase the risk of adverse events (e.g., dyspnea, fatigue) and reduce adherence.

The present study found that exercise programs with ≤3 times per week were associated with a larger pooled effect size compared with higher-frequency programs. This finding should be interpreted cautiously as an exploratory observation based on pooled effect sizes rather than statistically significant between-subgroup differences. This pattern may be partly explained by the need for adequate recovery time between exercise sessions in patients with COPD. Skeletal muscle endurance is significantly reduced in this population [58], and higher-frequency training may exacerbate muscle fatigue and impair adaptive responses to exercise. Moreover, from a behavioral perspective, lower-frequency programs are associated with higher adherence rates, as they impose a lower burden on patients’ daily lives [69].

Importantly, this observed pattern may also reflect structural confounding between exercise frequency and intervention type. Several interventions with higher frequency and higher weekly volume in the included studies were traditional Chinese exercise modalities, which are typically performed daily and differ from supervised aerobic or mixed exercise programs in terms of intensity progression, movement structure, and training objectives. Therefore, frequency in this context may not represent an independent training “dose” but is partly embedded within distinct intervention types, introducing potential confounding among moderator variables. This is critical, as adherence to exercise training is a key determinant of long-term success in pulmonary rehabilitation.

Subgroup analysis based on weekly time indicated that programs with ≤180 min per week were associated with a larger pooled effect size compared with higher-volume programs. This finding aligns with a previous meta-analysis, which reported that excessive weekly exercise volume is associated with increased fatigue and reduced adherence in COPD patients [69]. However, similar to exercise frequency, weekly volume is not independent of intervention type in the included evidence base, as traditional Chinese exercise programs were more frequently categorized into higher-frequency or higher-volume groups, whereas supervised aerobic or mixed programs tended to fall into lower or moderate weekly volume ranges. When combined with the subgroup findings on session duration and frequency, these results indicate that the observed patterns in effect estimates across subgroups are exploratory and likely reflect interrelated differences in intervention characteristics (type, structure, and training modality) rather than independent or causal effects of frequency or weekly volume alone. This design balances the need for sufficient exercise stimulus with the physiological limitations of the patient population.

Supervised exercise interventions were associated with a larger pooled effect estimate compared with unsupervised interventions, consistent with prior research [70]. The benefits of supervision can be attributed to several factors: first, trained professionals can individualize exercise intensity to ensure that patients train within a safe and effective range (e.g., 60–80% of heart rate reserve), optimizing the training stimulus while minimizing the risk of adverse events [71]. Second, supervised training ensures adherence to proper exercise technique, which reduces ineffective exertion and enhances the physiological benefits of training. Third, supervision improves exercise adherence by providing motivation, accountability, and real-time management of symptoms (e.g., dyspnea, fatigue) [72].

Despite these benefits, unsupervised programs remain a valuable alternative, particularly in regions with limited access to rehabilitation services, as they are more cost-effective and accessible [73]. However, it is important to note that unsupervised programs may be associated with lower adherence and less optimal outcomes in the long term, highlighting the need for structured remote monitoring and support (e.g., telephone check-ins, mobile health applications) to enhance their efficacy [74,75].

### 4.4. Limitations

This study has several limitations. First, there was considerable heterogeneity across the included studies in terms of exercise prescription as well as participant characteristics, which may have influenced the pooled estimates. Second, most studies assessed aerobic capacity using the 6MWD, whereas only a small number of studies reported VO_2_peak, a more direct and objective indicator of aerobic capacity. This imbalance in outcome reporting may limit the broader applicability and interpretability of the findings across different measures of aerobic performance. Third, most studies did not report detailed data on exercise intensity, which is a critical determinant of training efficacy; this precluded the performance of subgroup analyses based on intensity. Fourth, the inability to blind participants and personnel to exercise interventions introduces the potential for performance bias, which is inherent to exercise-based randomized controlled trials and may have influenced outcome estimates. Finally, the exclusion of non-English language studies may have introduced a degree of language bias; while this restriction is common in meta-analyses due to feasibility and resource constraints, it may limit the comprehensiveness of the evidence base and should be considered when interpreting the generalizability of the findings.

## 5. Conclusions

The present systematic review and meta-analysis provides robust evidence that exercise significantly improves aerobic capacity in patients with COPD, as measured by the 6MWD. Subgroup analyses suggest that certain exercise characteristics, including mixed exercise modalities, session durations of ≥60 min, weekly frequencies of ≤3 times, total weekly volumes of ≤180 min, and professional supervision, may be associated with more favorable outcomes. However, these patterns should be interpreted as exploratory and hypothesis-generating trends, as between-group differences and meta-regression analyses did not demonstrate statistically significant moderator effects.

These findings support the potential value of individualized exercise prescriptions tailored to the physiological limitations and preferences of COPD patients. Future research should focus on investigating the long-term effects of exercise training on clinical outcomes (e.g., hospital readmissions, mortality) and exploring the efficacy of technology-assisted remote exercise programs to improve access to pulmonary rehabilitation services.

## Data Availability

No new data were created or analyzed in this study. Data sharing is not applicable to this article.

## Supplementary Materials

The following supporting information can be downloaded at: https://www.mdpi.com/article/10.3390/healthcare14142165/s1, Figure S1: Results of Cochrane risk of bias tool; Figure S2: Funnel plot; Figure S3: Sensitivity analysis results; Table S1: Search strategies; Table S2: Characteristics of the included studies; Table S3: Results of meta-regression; Table S4: Results of Egger’s test; Table S5: GRADE summary of evidence.

## Abbreviations

The following abbreviations are used in this manuscript:

COPD	Chronic obstructive pulmonary disease
HRQOL	Health-related quality of life
PR	Pulmonary rehabilitation
RCT	Randomized controlled trial
PRISMA	Preferred Reporting Items for Systematic Reviews and Meta-Analyses
BMI	Body mass index
SD	Standard deviation
GRADE	Grading of Recommendations Assessment, Development and Evaluation
6MWD	6 min walk distance
WMD	Weighted mean difference
CI	Confidence interval
