# Supplementary material for: Effects of Exercise on Aerobic Capacity in People with Chronic Obstructive Pulmonary Disease: A Systematic Review and Meta-Analysis of Randomized Controlled Trials
Source: Healthcare (Basel). 2026 Jul 17;14(14):2165. doi: 10.3390/healthcare14142165 (PMC13410075; doi:10.3390/healthcare14142165)
Supplement: Supplementary file 1 [file healthcare-14-02165-s001.zip › healthcare-4354568-supplementary.pdf]

Supplemental material

Effects of exercise on aerobic capacity in people with chronic obstructive pulmonary disease: a systematic review and meta-analysis of randomized controlled trials

Figure S1. Results of Cochrane risk of bias tool..2

Figure S2. Funnel plot..3

Figure S3. Sensitivity analysis results...4

Table S1. Search strategies...5

Table S2. Characteristics of the included studies..16

Table S3. Results of meta-regression...21

Table S4. Results of Egger's test...22

Table S5. GRADE summary of evidence...23

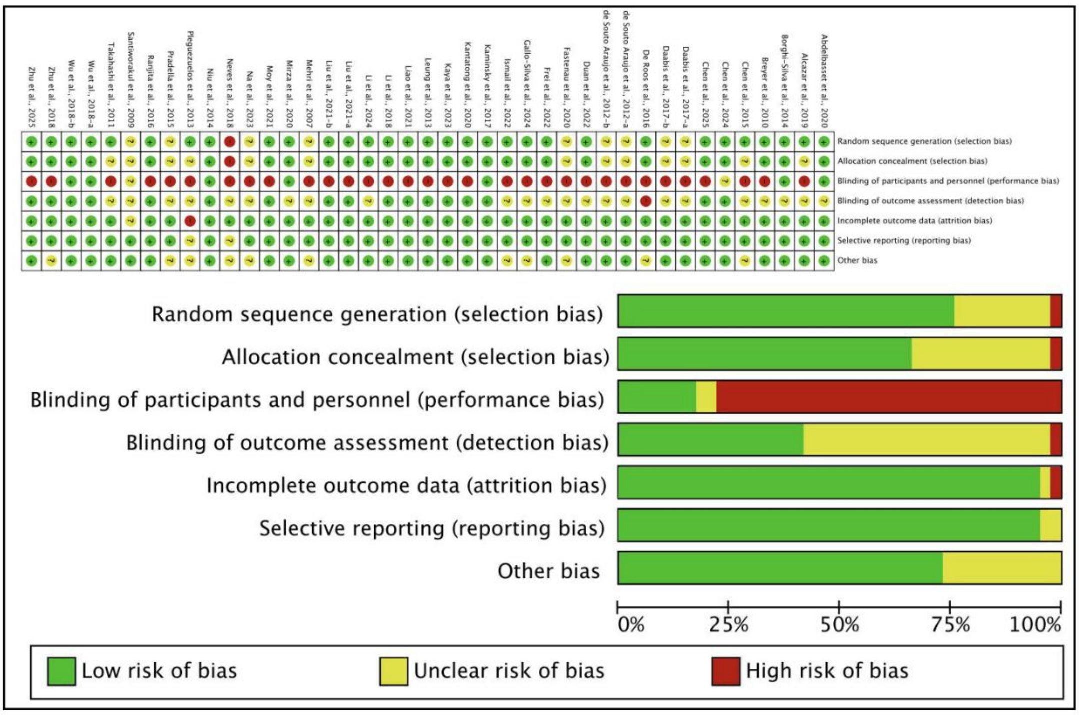

Figure S1. Results of Cochrane risk of bias tool.

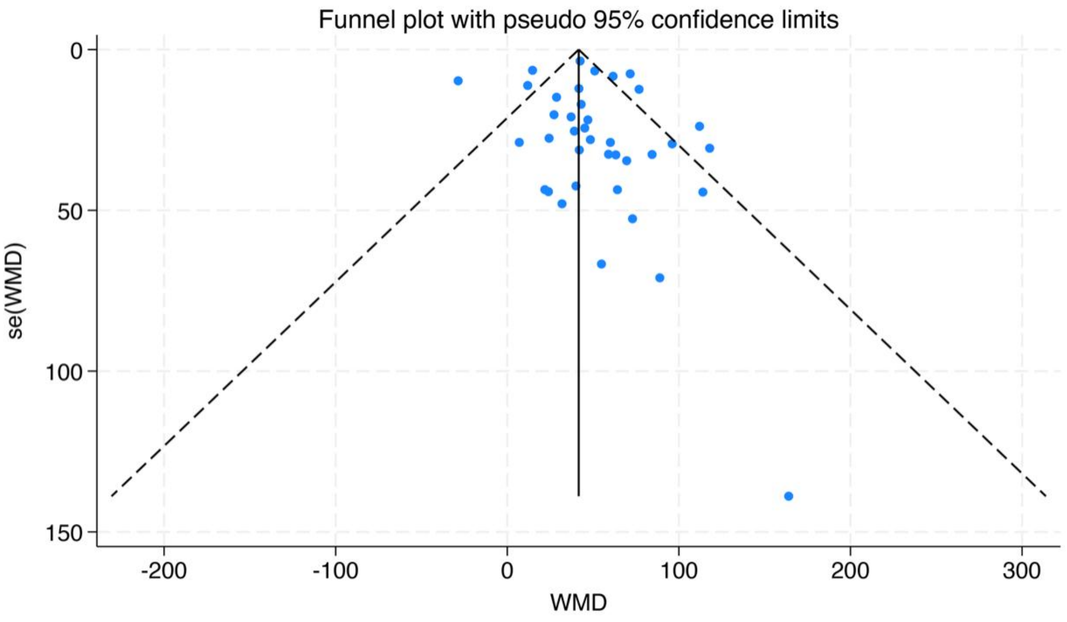

Figure S2. Funnel plot.

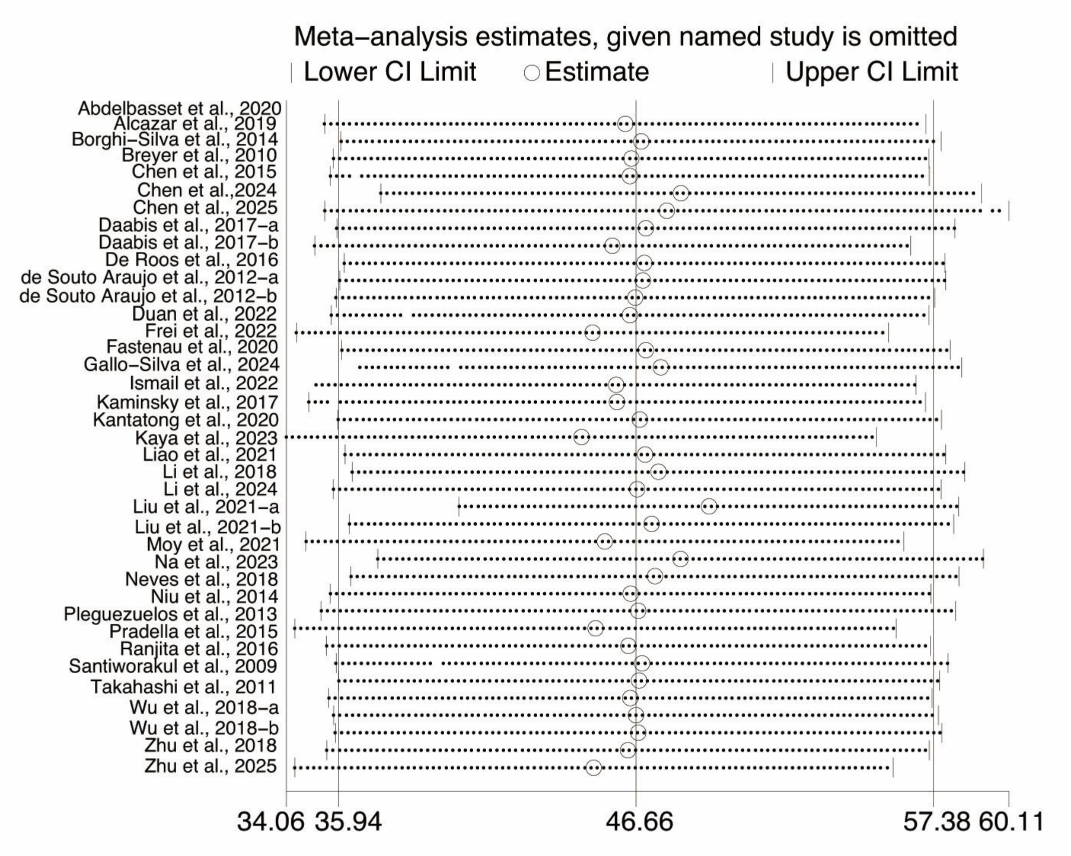

Figure S3. Sensitivity analysis results.

Table S1. Search strategies.

PubMed

Search Component	Search Number	Search Query
Exercise Terms	#1	(Physical exercise programs[Title/Abstract]) OR (Physical Therapy Modalities[Title/Abstract])) OR (Physical Therapy Modalities[Title/Abstract])) OR (Modalities, Physical Therapy[Title/Abstract])) OR (Modality, Physical Therapy[Title/Abstract])) OR (Physical Therapy Modality[Title/Abstract])) OR (Physiotherapy (Techniques[Title/Abstract]))) OR (Physiotherapies (Techniques[Title/Abstract]))) OR (Physical Therapy Techniques[Title/Abstract])) OR (Physical Therapy Technique[Title/Abstract])) OR (Techniques, Physical Therapy[Title/Abstract])) OR (Exercise Movement Techniques[Title/Abstract])) OR (Exercise Movement Techniques[Title/Abstract])) OR (Movement Techniques, Exercise[Title/Abstract])) OR (Exercise Movement Technics[Title/Abstract])) OR (Exercise Therapy[Title/Abstract])) OR (Exercise Therapy[Title/Abstract])) OR (Therapy, Exercise[Title/Abstract])) OR (Exercise Therapies[Title/Abstract])) OR (Therapies, Exercise[Title/Abstract])) OR (Exercise[Title/Abstract])) OR (Exercise[Title/Abstract])) OR (Exercises[Title/Abstract])) OR (Exercise, Physical[Title/Abstract])) OR (Exercises, Physical[Title/Abstract])) OR (Physical Exercise[Title/Abstract])) OR (Physical Exercises[Title/Abstract])) OR (Exercise, Isometric[Title/Abstract])) OR (Exercises, Isometric[Title/Abstract])) OR (Isometric Exercises[Title/Abstract])) OR (Isometric Exercise[Title/Abstract])) OR (Exercise, Aerobic[Title/Abstract])) OR (Aerobic Exercises[Title/Abstract])) OR (Aerobic Exercise[Title/Abstract])) OR (Resistance Training[Title/Abstract])) OR (Resistance Training[Title/Abstract])) OR (Training, Resistance[Title/Abstract])) OR (Strength Training[Title/Abstract])) OR (Training, Strength[Title/Abstract])) OR (Weight-Lifting Strengthening Program[Title/Abstract])) OR (Strengthening Program, Weight-Lifting[Title/Abstract])) OR (Strengthening Programs, Weight-Lifting[Title/Abstract])) OR (Weight Lifting Strengthening Program[Title/Abstract])) OR (Weight-Lifting Strengthening Programs[Title/Abstract])) OR (Weight-Lifting

		Exercise Program[Title/Abstract])) OR (Exercise Program, Weight-Lifting[Title/Abstract])) OR (Exercise Programs, Weight-Lifting[Title/Abstract])) OR (Weight Lifting Exercise Program[Title/Abstract])) OR (Weight-Lifting Exercise Programs[Title/Abstract])) OR (Weight-Bearing Strengthening Program[Title/Abstract])) OR (Strengthening Program, Weight-Bearing[Title/Abstract])) OR (Strengthening Programs, Weight-Bearing[Title/Abstract])) OR (Weight Bearing Strengthening Program[Title/Abstract])) OR (Weight-Bearing Strengthening Programs[Title/Abstract])) OR (Weight-Bearing Exercise Program[Title/Abstract])) OR (Exercise Program, Weight-Bearing[Title/Abstract])) OR (Exercise Programs, Weight-Bearing[Title/Abstract])) OR (Weight Bearing Exercise Program[Title/Abstract])) OR (Weight-Bearing Exercise Programs[Title/Abstract])) OR (Physical Activity[Title/Abstract])) OR (Activities, Physical[Title/Abstract])) OR (Activity, Physical[Title/Abstract])) OR (Physical Activities[Title/Abstract])) OR (Acute Exercise[Title/Abstract])) OR (Acute Exercises[Title/Abstract])) OR (Exercise, Acute[Title/Abstract])) OR (Exercises, Acute[Title/Abstract])) OR (Exercise Training[Title/Abstract])) OR (Exercise Trainings[Title/Abstract])) OR (Training, Exercise[Title/Abstract])) OR (Trainings, Exercise[Title/Abstract])) OR (Exercises, Aerobic[Title/Abstract])
Aerobic capacity Terms	#2	(Aerobic capacity[Title/Abstract]) OR (Maximum oxygen consumption[Title/Abstract])) OR (VO2max[Title/Abstract])) OR (peak oxygen consumption[Title/Abstract])) OR (peak VO2[Title/Abstract])) OR (6-minute walk distance[Title/Abstract])) OR (6MWD[Title/Abstract])) OR (6-minute walk test[Title/Abstract])) OR (6MWT[Title/Abstract])
COPD Terms	#3	(chronic obstructive pulmonary disease[Title/Abstract]) OR (Pulmonary Disease, Chronic Obstructive[Title/Abstract])) OR (Chronic Obstructive Lung Disease[Title/Abstract])) OR (Chronic Obstructive Pulmonary Diseases[Title/Abstract])) OR (COAD[Title/Abstract])) OR (COPD[Title/Abstract])) OR (Chronic Obstructive Airway Disease[Title/Abstract])) OR (Chronic Obstructive Pulmonary Disease[Title/Abstract])) OR (Airflow Obstruction, Chronic[Title/Abstract])) OR (Airflow Obstructions, Chronic[Title/Abstract])) OR (Chronic Airflow Obstructions[Title/Abstract])) OR (Chronic Airflow Obstruction[Title/Abstract])

Combined Search	#4	#1 AND #2 AND #3

Web of Science

Search Component	Search Number	Search Query
Exercise Terms	#1	(TI =(exercise) OR TI =(Exercises) OR TI =(Physical Activity) OR TI =(Activities, Physical) OR TI =(Activity, Physical) OR TI =(Physical Activities) OR TI =(Exercise, Physical) OR TI =(Exercises, Physical) OR TI =(Physical Exercise) OR TI =(Physical Exercises) OR TI =(Acute Exercise) OR TI =(Acute Exercises) OR TI =(Exercise, Acute) OR TI =(Exercises, Acute) OR TI =(Exercise, Isometric) OR TI =(Exercises, Isometric) OR TI =(Isometric Exercises) OR TI =(Isometric Exercise) OR TI =(Exercise, Aerobic) OR TI =(Aerobic Exercise) OR TI =(Aerobic Exercises) OR TI =(Exercises, Aerobic) OR TI =(Exercise Training) OR TI =(Exercise Trainings) OR TI =(Training, Exercise) OR TI =(Trainings, Exercise) OR TI =(Physical exercise programs) OR TI =(Physical Therapy Modalities) OR TI =(Modalities, Physical Therapy) OR TI =(Modality, Physical Therapy) OR TI =(Physical Therapy Modality) OR TI =(Physiotherapy (Techniques)) OR TI =(Physiotherapies (Techniques)) OR TI =(Physical Therapy Techniques) OR TI =(Physical Therapy Technique) OR TI =(Techniques, Physical Therapy) OR TI =(Exercise Movement Techniques) OR TI =(Movement Techniques, Exercise) OR TI =(Exercise Movement Technics) OR TI =(Exercise Therapy) OR TI =(Therapy, Exercise) OR TI =(Exercise Therapies) OR TI =(Therapies, Exercise) OR TI =(Resistance Training) OR TI =(Training, Resistance) OR TI =(Strength Training) OR TI =(Training, Strength) OR TI =(Weight-Lifting Strengthening Program) OR TI =(Strengthening Program, Weight-Lifting) OR TI =(Strengthening Programs, Weight-Lifting) OR TI =(Weight Lifting Strengthening Program) OR TI =(Weight-Lifting Strengthening Programs) OR TI =(Weight-Lifting Exercise Program) OR TI =(Exercise Program, Weight-Lifting) OR TI =(Exercise Programs, Weight-Lifting) OR TI =(Weight Lifting Exercise Program) OR TI =(Weight-Lifting Exercise Programs) OR TI =(Weight-Bearing Strengthening Program) OR TI =(Strengthening Program, Weight-Bearing) OR TI

		=(Strengthening Programs, Weight-Bearing) OR TI =(Weight Bearing Strengthening Program) OR TI =(Weight-Bearing Strengthening Programs) OR TI =(Weight-Bearing Exercise Program) OR TI =(Exercise Program, Weight-Bearing) OR TI =(Exercise Programs, Weight-Bearing) OR TI =(Weight Bearing Exercise Program) OR TI =(Weight-Bearing Exercise Programs) OR AB =(exercise) OR AB =(Exercises) OR AB =(Physical Activity) OR AB =(Activities, Physical) OR AB =(Activity, Physical) OR AB =(Physical Activities) OR AB =(Exercise, Physical) OR AB =(Exercises, Physical) OR AB =(Physical Exercise) OR AB =(Physical Exercises) OR AB =(Acute Exercise) OR AB =(Acute Exercises) OR AB =(Exercise, Acute) OR AB =(Exercises, Acute) OR AB =(Exercise, Isometric) OR AB =(Exercises, Isometric) OR AB =(Isometric Exercises) OR AB =(Isometric Exercise) OR AB =(Exercise, Aerobic) OR AB =(Aerobic Exercise) OR AB =(Aerobic Exercises) OR AB =(Exercises, Aerobic) OR AB =(Exercise Training) OR AB =(Exercise Trainings) OR AB =(Training, Exercise) OR AB =(Trainings, Exercise) OR AB =(Physical exercise programs) OR AB =(Physical Therapy Modalities) OR AB =(Modalities, Physical Therapy) OR AB =(Modality, Physical Therapy) OR AB =(Physical Therapy Modality) OR AB =(Physiotherapy (Techniques)) OR AB =(Physiotherapies (Techniques)) OR AB =(Physical Therapy Techniques) OR AB =(Physical Therapy Technique) OR AB =(Techniques, Physical Therapy) OR AB =(Exercise Movement Techniques) OR AB =(Movement Techniques, Exercise) OR AB =(Exercise Movement Technics) OR AB =(Exercise Therapy) OR AB =(Therapy, Exercise) OR AB =(Exercise Therapies) OR AB =(Therapies, Exercise) OR AB =(Resistance Training) OR AB =(Training, Resistance) OR AB =(Strength Training) OR AB =(Training, Strength) OR AB =(Weight-Lifting Strengthening Program) OR AB =(Strengthening Program, Weight-Lifting) OR AB =(Strengthening Programs, Weight-Lifting) OR AB =(Weight Lifting Strengthening Program) OR AB =(Weight-Lifting Strengthening Programs) OR AB =(Weight-Lifting Exercise Program) OR AB =(Exercise Program, Weight-Lifting) OR AB =(Exercise Programs, Weight-Lifting) OR AB =(Weight Lifting Exercise Program) OR AB =(Weight-Lifting Exercise Programs) OR AB =(Weight-Bearing Strengthening Program) OR AB =(Strengthening Program, Weight-Bearing) OR AB =(Strengthening Programs, Weight-Bearing) OR AB =(Weight

		Bearing Strengthening Program) OR AB =(Weight-Bearing Strengthening Programs) OR AB =(Weight-Bearing Exercise Program) OR AB =(Exercise Program, Weight-Bearing) OR AB =(Exercise Programs, Weight-Bearing) OR AB =(Weight Bearing Exercise Program) OR AB =(Weight-Bearing Exercise Programs))
Aerobic capacity Terms	#2	TI =(Aerobic capacity) OR TI =(Maximum oxygen consumption) OR TI =(VO2max) OR TI =(peak oxygen consumption) OR TI =(peak VO2) OR TI =(6-minute walk distance) OR TI =(6MWD) OR TI =(6-minute walk test) OR TI =(6MWT) OR AB =(Aerobic capacity) OR AB =(Maximum oxygen consumption) OR AB =(VO2max) OR AB =(peak oxygen consumption) OR AB =(peak VO2) OR AB =(6-minute walk distance) OR AB =(6MWD) OR AB =(6-minute walk test) OR AB =(6MWT)
COPD Terms	#3	TI =(chronic obstructive pulmonary disease) OR TI =(Pulmonary Disease, Chronic Obstructive) OR TI =(Chronic Obstructive Lung Disease) OR TI =(Chronic Obstructive Pulmonary Diseases) OR TI =(COAD) OR TI =(COPD) OR TI =(Chronic Obstructive Airway Disease) OR TI =(Chronic Obstructive Pulmonary Disease) OR TI =(Airflow Obstruction, Chronic) OR TI =(Airflow Obstructions, Chronic) OR TI =(Chronic Airflow Obstructions) OR TI =(Chronic Airflow Obstruction) OR AB =(chronic obstructive pulmonary disease) OR AB =(Pulmonary Disease, Chronic Obstructive) OR AB =(Chronic Obstructive Lung Disease) OR AB =(Chronic Obstructive Pulmonary Diseases) OR AB =(COAD) OR AB =(COPD) OR AB =(Chronic Obstructive Airway Disease) OR AB =(Chronic Obstructive Pulmonary Disease) OR AB =(Airflow Obstruction, Chronic) OR AB =(Airflow Obstructions, Chronic) OR AB =(Chronic Airflow Obstructions) OR AB =(Chronic Airflow Obstruction)
Combined Search	#4	#1 AND #2 AND #3

Cochrane Library

Search Component	Search Number	Search Query
Exercise Terms	#1	(Physical exercise programs):ti,ab,kw OR (Physical Therapy Modalities):ti,ab,kw OR (Physical Therapy Modalities):ti,ab,kw OR (Modalities, Physical Therapy):ti,ab,kw OR (Modality,

		Physical Therapy):ti,ab,kw OR (Physical Therapy Modality):ti,ab,kw OR (Physiotherapy Techniques):ti,ab,kw OR (Physiotherapies Techniques):ti,ab,kw OR (Physical Therapy Techniques):ti,ab,kw OR (Physical Therapy Technique):ti,ab,kw OR (Techniques, Physical Therapy):ti,ab,kw OR (Exercise Movement Techniques):ti,ab,kw OR (Exercise Movement Techniques):ti,ab,kw OR (Movement Techniques, Exercise):ti,ab,kw OR (Exercise Movement Technics):ti,ab,kw OR (Exercise Therapy):ti,ab,kw OR (Exercise Therapy):ti,ab,kw OR (Therapy, Exercise):ti,ab,kw OR (Exercise Therapies):ti,ab,kw OR (Therapies, Exercise):ti,ab,kw OR (Exercise):ti,ab,kw OR (Exercises):ti,ab,kw OR (Exercise, Physical):ti,ab,kw OR (Exercises, Physical):ti,ab,kw OR (Physical Exercise):ti,ab,kw OR (Physical Exercises):ti,ab,kw OR (Exercise, Isometric):ti,ab,kw OR (Exercises, Isometric):ti,ab,kw OR (Isometric Exercises):ti,ab,kw OR (Isometric Exercise):ti,ab,kw OR (Exercise, Aerobic):ti,ab,kw OR (Aerobic Exercises):ti,ab,kw OR (Exercises, Aerobic):ti,ab,kw OR (Aerobic Exercise):ti,ab,kw OR (Resistance Training):ti,ab,kw OR (Training, Resistance):ti,ab,kw OR (Strength Training):ti,ab,kw OR (Training, Strength):ti,ab,kw OR (Weight-Lifting Strengthening Program):ti,ab,kw OR (Strengthening Program, Weight-Lifting):ti,ab,kw OR (Weight Lifting Strengthening Program):ti,ab,kw OR (Weight-Lifting Strengthening Programs):ti,ab,kw OR (Weight-Lifting Exercise Program):ti,ab,kw OR (Exercise Program, Weight-Lifting):ti,ab,kw OR (Exercise Programs, Weight-Lifting):ti,ab,kw OR (Weight Lifting Exercise Program):ti,ab,kw OR (Weight-Lifting Exercise Programs):ti,ab,kw OR (Weight-Bearing Strengthening Program):ti,ab,kw OR (Strengthening Program, Weight-Bearing):ti,ab,kw OR (Strengthening Programs, Weight-Bearing):ti,ab,kw OR (Weight Bearing Strengthening Program):ti,ab,kw OR (Weight-Bearing Strengthening Programs):ti,ab,kw OR (Weight-Bearing Exercise Program):ti,ab,kw OR (Exercise Program, Weight-Bearing):ti,ab,kw OR (Exercise Programs, Weight-Bearing):ti,ab,kw OR (Weight Bearing Exercise Program):ti,ab,kw OR (Weight-Bearing Exercise Programs):ti,ab,kw OR (Physical Activity):ti,ab,kw OR (Activities, Physical):ti,ab,kw OR (Activity, Physical):ti,ab,kw

		OR (Physical Activities):ti,ab,kw OR (Acute Exercise):ti,ab,kw OR (Acute Exercises):ti,ab,kw OR (Exercise, Acute):ti,ab,kw OR (Exercises, Acute):ti,ab,kw OR (Exercise Training):ti,ab,kw OR (Exercise Trainings):ti,ab,kw OR (Training, Exercise):ti,ab,kw OR (Trainings, Exercise):ti,ab,kw
Aerobic capacity Terms	#2	(Aerobic capacity):ti,ab,kw OR (Maximum oxygen consumption):ti,ab,kw OR (VO2max):ti,ab,kw OR (peak oxygen consumption):ti,ab,kw OR (peak VO2):ti,ab,kw OR (6-minute walk distance):ti,ab,kw OR (6MWD):ti,ab,kw OR (6-minute walk test):ti,ab,kw OR (6MWT):ti,ab,kw
COPD Terms	#3	(chronic obstructive pulmonary disease):ti,ab,kw OR (Pulmonary Disease, Chronic Obstructive):ti,ab,kw OR (Chronic Obstructive Lung Disease):ti,ab,kw OR (Chronic Obstructive Pulmonary Diseases):ti,ab,kw OR (COAD):ti,ab,kw OR (COPD):ti,ab,kw OR (Chronic Obstructive Airway Disease):ti,ab,kw OR (Chronic Obstructive Pulmonary Disease):ti,ab,kw OR (Airflow Obstruction, Chronic):ti,ab,kw OR (Airflow Obstructions, Chronic):ti,ab,kw OR (Chronic Airflow Obstructions):ti,ab,kw OR (Chronic Airflow Obstruction):ti,ab,kw
Combined Search	#4	#1 AND #2 AND #3

Scopus

Search Component	Search Number	Search Query
Exercise Terms	#1	(TITLE-ABS-KEY(exercise) OR TITLE-ABS-KEY(Exercises) OR TITLE-ABS-KEY(Physical Activity) OR TITLE-ABS-KEY(Activities, Physical) OR TITLE-ABS-KEY(Activity, Physical) OR TITLE-ABS-KEY(Physical Activities) OR TITLE-ABS-KEY(Exercise, Physical) OR TITLE-ABS-KEY(Exercises, Physical) OR TITLE-ABS-KEY(Physical Exercise) OR TITLE-ABS-KEY(Physical Exercises) OR TITLE-ABS-KEY(Acute Exercise) OR TITLE-ABS-KEY(Acute Exercises) OR TITLE-ABS-KEY(Exercise, Acute) OR TITLE-ABS-KEY(Exercises, Acute) OR TITLE-ABS-KEY(Exercise, Isometric) OR TITLE-ABS-KEY(Exercises, Isometric) OR

		TITLE-ABS-KEY(Isometric Exercises) OR TITLE-ABS-KEY(Isometric Exercise) OR TITLE-ABS-KEY(Exercise, Aerobic) OR TITLE-ABS-KEY(Aerobic Exercise) OR TITLE-ABS-KEY(Aerobic Exercises) OR TITLE-ABS-KEY(Exercises, Aerobic) OR TITLE-ABS-KEY(Exercise Training) OR TITLE-ABS-KEY(Exercise Trainings) OR TITLE-ABS-KEY(Training, Exercise) OR TITLE-ABS-KEY(Trainings, Exercise) OR TITLE-ABS-KEY(Physical exercise programs) OR TITLE-ABS-KEY(Physical Therapy Modalities) OR TITLE-ABS-KEY(Modalities, Physical Therapy) OR TITLE-ABS-KEY(Modality, Physical Therapy) OR TITLE-ABS-KEY(Physical Therapy Modality) OR TITLE-ABS-KEY(Physiotherapy Techniques) OR TITLE-ABS-KEY(Physiotherapies Techniques) OR TITLE-ABS-KEY(Physical Therapy Techniques) OR TITLE-ABS-KEY(Physical Therapy Technique) OR TITLE-ABS-KEY(Techniques, Physical Therapy) OR TITLE-ABS-KEY(Exercise Movement Techniques) OR TITLE-ABS-KEY(Movement Techniques, Exercise) OR TITLE-ABS-KEY(Exercise Movement Technics) OR TITLE-ABS-KEY(Exercise Therapy) OR TITLE-ABS-KEY(Therapy, Exercise) OR TITLE-ABS-KEY(Exercise Therapies) OR TITLE-ABS-KEY(Therapies, Exercise) OR TITLE-ABS-KEY(Resistance Training) OR TITLE-ABS-KEY(Training, Resistance) OR TITLE-ABS-KEY(Strength Training) OR TITLE-ABS-KEY(Training, Strength) OR TITLE-ABS-KEY(Weight-Lifting Strengthening Program) OR TITLE-ABS-KEY(Strengthening Program, Weight-Lifting) OR TITLE-ABS-KEY(Strengthening Programs, Weight-Lifting) OR TITLE-ABS-KEY(Weight Lifting Strengthening Program) OR TITLE-ABS-KEY(Weight-Lifting Strengthening Programs) OR TITLE-ABS-KEY(Weight-Lifting Exercise Program) OR TITLE-ABS-KEY(Exercise Program, Weight-Lifting) OR TITLE-ABS-KEY(Exercise Programs, Weight-Lifting) OR TITLE-ABS-KEY(Weight Lifting Exercise Program) OR TITLE-ABS-KEY(Weight-Lifting Exercise Programs) OR TITLE-ABS-KEY(Weight-Bearing Strengthening Program) OR TITLE-ABS-KEY(Strengthening Program, Weight-Bearing)

		OR TITLE-ABS-KEY(Strengthening Programs, Weight-Bearing) OR TITLE-ABS-KEY(Weight Bearing Strengthening Program) OR TITLE-ABS-KEY(Weight-Bearing Strengthening Programs) OR TITLE-ABS-KEY(Weight-Bearing Exercise Program) OR TITLE-ABS-KEY(Exercise Program, Weight-Bearing) OR TITLE-ABS-KEY(Exercise Programs, Weight-Bearing) OR TITLE-ABS-KEY(Weight Bearing Exercise Program) OR TITLE-ABS-KEY(Weight-Bearing Exercise Programs))
Aerobic capacity Terms	#2	(TITLE-ABS-KEY(Aerobic capacity) OR TITLE-ABS-KEY(Maximum oxygen consumption) OR TITLE-ABS-KEY(VO2max) OR TITLE-ABS-KEY(peak oxygen consumption) OR TITLE-ABS-KEY(peak VO2) OR TITLE-ABS-KEY(6-minute walk distance) OR TITLE-ABS-KEY(6MWD) OR TITLE-ABS-KEY(6-minute walk test) OR TITLE-ABS-KEY(6MWT))
COPD Terms	#3	(TITLE-ABS-KEY(chronic obstructive pulmonary disease) OR TITLE-ABS-KEY(Pulmonary Disease, Chronic Obstructive) OR TITLE-ABS-KEY(Chronic Obstructive Lung Disease) OR TITLE-ABS-KEY(Chronic Obstructive Pulmonary Diseases) OR TITLE-ABS-KEY(COAD) OR TITLE-ABS-KEY(COPD) OR TITLE-ABS-KEY(Chronic Obstructive Airway Disease) OR TITLE-ABS-KEY(Chronic Obstructive Pulmonary Disease) OR TITLE-ABS-KEY(Airflow Obstruction, Chronic) OR TITLE-ABS-KEY(Airflow Obstructions, Chronic) OR TITLE-ABS-KEY(Chronic Airflow Obstructions) OR TITLE-ABS-KEY(Chronic Airflow Obstruction)
Combined Search	#4	#1 AND #2 AND #3

Embase

Search Component	Search Number	Search Query
Exercise Terms	#1	'exercise':ab,ti OR 'Exercises':ab,ti OR 'Physical Activity':ab,ti OR 'Activities, Physical':ab,ti OR 'Activity, Physical':ab,ti OR 'Physical Activities':ab,ti OR 'Exercise, Physical':ab,ti OR 'Exercises, Physical':ab,ti OR 'Physical Exercise':ab,ti OR 'Physical Exercises':ab,ti OR 'Acute Exercise':ab,ti OR 'Acute Exercises':ab,ti OR 'Exercise, Acute':ab,ti OR 'Exercises, Acute':ab,ti OR 'Exercise, Isometric':ab,ti OR 'Exercises, Isometric':ab,ti OR 'Isometric Exercises':ab,ti OR 'Isometric Exercise':ab,ti OR 'Exercise, Aerobic':ab,ti OR

		'Aerobic Exercise':ab,ti OR 'Aerobic Exercises':ab,ti OR 'Exercises, Aerobic':ab,ti OR 'Exercise Training':ab,ti OR 'Exercise Trainings':ab,ti OR 'Training, Exercise':ab,ti OR 'Trainings, Exercise':ab,ti OR 'Physical exercise programs':ab,ti OR 'Physical Therapy Modalities':ab,ti OR 'Modalities, Physical Therapy':ab,ti OR 'Modality, Physical Therapy':ab,ti OR 'Physical Therapy Modality':ab,ti OR 'Physiotherapy (Techniques)':ab,ti OR 'Physiotherapies (Techniques)':ab,ti OR 'Physical Therapy Techniques':ab,ti OR 'Physical Therapy Technique':ab,ti OR 'Techniques, Physical Therapy':ab,ti OR 'Exercise Movement Techniques':ab,ti OR 'Movement Techniques, Exercise':ab,ti OR 'Exercise Movement Technics':ab,ti OR 'Exercise Therapy':ab,ti OR 'Therapy, Exercise':ab,ti OR 'Exercise Therapies':ab,ti OR 'Therapies, Exercise':ab,ti OR 'Resistance Training':ab,ti OR 'Training, Resistance':ab,ti OR 'Strength Training':ab,ti OR 'Training, Strength':ab,ti OR 'Weight-Lifting Strengthening Program':ab,ti OR 'Strengthening Program, Weight-Lifting':ab,ti OR 'Strengthening Programs, Weight-Lifting':ab,ti OR 'Weight Lifting Strengthening Program':ab,ti OR 'Weight-Lifting Strengthening Programs':ab,ti OR 'Weight-Lifting Exercise Program':ab,ti OR 'Exercise Program, Weight-Lifting':ab,ti OR 'Exercise Programs, Weight-Lifting':ab,ti OR 'Weight Lifting Exercise Program':ab,ti OR 'Weight-Lifting Exercise Programs':ab,ti OR 'Weight-Bearing Strengthening Program':ab,ti OR 'Strengthening Program, Weight-Bearing':ab,ti OR 'Strengthening Programs, Weight-Bearing':ab,ti OR 'Weight Bearing Strengthening Program':ab,ti OR 'Weight-Bearing Strengthening Programs':ab,ti OR 'Weight-Bearing Exercise Program':ab,ti OR 'Exercise Program, Weight-Bearing':ab,ti OR 'Exercise Programs, Weight-Bearing':ab,ti OR 'Weight Bearing Exercise Program':ab,ti OR 'Weight-Bearing Exercise Programs':ab,ti
Aerobic capacity Terms	#2	'Aerobic capacity':ab,ti OR ' Maximum oxygen consumption':ab,ti OR 'VO2max':ab,ti OR ' peak oxygen consumption':ab,ti OR 'peak VO2':ab,ti OR '6-minute walk distance':ab,ti OR '6MWD':ab,ti OR '6-minute walk test':ab,ti OR '6MWT':ab,ti
COPD Terms	#3	'chronic obstructive pulmonary disease':ab,ti OR 'Pulmonary Disease, Chronic Obstructive':ab,ti OR 'Chronic Obstructive Lung Disease':ab,ti OR 'Chronic Obstructive Pulmonary Diseases':ab,ti OR 'COAD':ab,ti OR 'COPD':ab,ti OR 'Chronic

		Obstructive Airway Disease':ab,ti OR 'Chronic Obstructive Pulmonary Disease':ab,ti OR 'Airflow Obstruction, Chronic':ab,ti OR 'Airflow Obstructions, Chronic':ab,ti OR 'Chronic Airflow Obstructions':ab,ti OR 'Chronic Airflow Obstruction':ab,ti
Combined Search	#4	#1 AND #2 AND #3

Table S2. Characteristics of the included studies.

Study	Sample size	Gender (M/F)	Age (years)	Severity	Intervention	Session duration	Frequency (times/week)	Duration (weeks)	Weekly time (min)	Supervision status	Indicators
Abdelbasset et al., 2020	Int: 20<br>Con: 20	Int:16/4<br>Con:13/7	Int: 71.1 ± 1.12<br>Con: 71.5 ± 1.2	FEV: 50-79%	Bicycle ergometer and arm crank exercise	14-34 min	3	12	100	Supervised	6MWD
Alcazar et al., 2019	Int: 14<br>Con: 15	Int: 11/3<br>Con: 13/2	Int:77.7 ± 7.9<br>Con:79.8 ± 6.4	NR	HIIT + Strength training	NR	2	12	NR	Supervised	6MWD
Borghi-Silva et al., 2014	Int: 10<br>Con: 10	Int: 7/3<br>Con: 5/5	Int: 67 ± 7<br>Con: 66 ± 10	NR	Aerobic exercise	35 min	3	12	105	Supervised	6MWD
Breyer et al., 2010	Int: 30<br>Con: 30	Int: 14/16<br>Con: 13/17	Int: 61.9 ± 8.87<br>Con: 59.0 ± 8.02	NR	Nordic walking	60 min	3	12	180	Supervised	6MWD
Chen et al., 2015	Int: 30<br>Con: 30	Int: 18/12<br>Con: 17/13	Int: 57.6 ± 7.6<br>Con: 54.2 ± 6.5	Grade II-III pulmonary function	Daoyin technique	NR	5	12	NR	Unsupervised	6MWD
Chen et al., 2024	Int: 158<br>Con: 160	Int: 89/69<br>Con: 88/72	Int: 61.52 ± 10.31<br>Con: 61.97 ± 10.91	Grade II-III pulmonary function	Baduanjin	30 min	10	24	300	Unsupervised	6MWD
Chen et al., 2025	Int: 32<br>Con: 31	Int: 21/11<br>Con: 24/7	Int: 69.03 ± 1.05<br>Con: 67.10 ± 1.20	Grade I-III pulmonary function	Traditional Chinese medicine exercises	45 min	4	12	180	Supervised	6MWD

Daabis et al., 2017	Int1: 15<br>Int2: 15<br>Con: 15	NR	Int1: 61 ± 8<br>Int2: 58 ± 7<br>Con: 60 ± 8	Acute exacerbation	Int1: Endurance training<br>Int2: Combined endurance and strength training	60 min	3	8	180	Supervised	6MWD
De Roos et al., 2016	Int: 21<br>Con: 24	Int: 8/18<br>Con: 10/16	Int: 69.4 ± 9.7<br>Con: 71.0 ± 9.4	Acute exacerbation	Integration of sports training and home exercise	60 min	2	10	120	Supervised	6MWD
de Souto Araujo et al., 2012	Int: 13<br>Con: 11	Int1: 5/8<br>Int2: 4/4<br>Con: 3/8	Int1: 56.9 ± 7.9<br>Int2: 62.4 ± 9.9<br>Con: 71.1 ± 10.1	GOLD stage II, 50% ≤ FEV1 < 80%	Combined endurance and strength training	90 min	3	8	270	Supervised	6MWD
Duan et al., 2022	Int: 39<br>Con: 40	Int: 21/15<br>Con: 20/14	Int: 72.27 ± 7.16<br>Con: 72.67 ± 7.21	Moderate to very severe	TBRS recumbent stepper	30 min	3	12	105	Supervised	6MWD
Fastenau et al., 2020	Int: 39<br>Con: 32	Int: 27/19<br>Con: 17/27	Int: 62.4 ± 9.1<br>Con: 62.6 ± 10.8	Moderate to very severe	Home exercise	60-90 min	2	4	120-180	Supervised	6MWD
Frei et al., 2022	Int: 61<br>Con: 62	Int: 30/31<br>Con: 32/30	Int: 66.1 ± 8.3<br>Con: 67.4 ± 7.9	COPD stage II-II	Home exercise	20 min	6	12	120	Unsupervised	6MWD
Gallo-Silva et al., 2024	Int: 11<br>Con: 11	Int: 11/0<br>Con: 11/0	Int: 65.5 ± 6.3<br>Con: 66.3 ± 10.2	Mild to moderate	Aerobic Training	60 min	3	8	180	Supervised	6MWD
Ismail et al., 2022	Int: 20<br>Con: 20	NR	Int: 60.35 ± 4.35<br>Con: 59.35 ± 3.82	COPD stages II-IV	Home exercise	110 min	3	8	330	Supervised	6MWD

Kaminsky et al., 2017	Int: 21<br>Con: 22	Int: 7/14<br>Con: 10/12	Int: 68 ± 7<br>Con: 68 ± 9	NR	Yoga breathing	60 min	3	12	NR	Mixed	6MWD
Kantatong et al., 2020	Int: 25<br>Con: 25	Int: 15/10<br>Con: 19/6	Int: 69.68 ± 7.67<br>Con: 67.48 ± 10.17	NR	Tai Chi Qigong	NR	4	24	NR	Mixed	6MWD
Kaya et al., 2023	Int: 12<br>Con: 12	Int: 10/2<br>Con: 10/2	Int: 65.17 ± 6.35<br>Con: 64.75 ± 8.49	mMRC score > 2, FEV1/FVC < 0.7, FEV1 < 80%	Creative dance	45 min	2	8	90	Supervised	6MWD
Leung et al., 2013	Int: 22<br>Con: 20	NR	NR	Mild to moderate	Tai Chi	60 min	2	12	120	Supervised	ESW
Liao et al., 2021	Int: 34<br>Con: 36	Int: 28/6<br>Con: 27/9	Int: 61.83 ± 6.63<br>Con: 61.21 ± 7.38	FEV1/FVC < 70%	Liu Zi Jue	30 min	14	12	420	Unsupervised	6MWD
Li et al., 2018	Int: 17<br>Con: 19	Int: 14/3<br>Con: 14/5	Int: 66 ± 9<br>Con: 66 ± 9	NR	Liu Zi Jue	60 min	6	24	360	Mixed	6MWD
Li et al., 2024	Int: 114<br>Con: 114	Int: 103/11<br>Con: 99/15	Int: 67.34 ± 7.45<br>Con: 67.63 ± 7.31	Acute worsening	Prone Daoyin technique	20 min	14	1	280	Supervised	6MWD
Liu et al., 2021	Int1: 15<br>Int2: 14<br>Con: 16	Int1: 12/3<br>Int2: 9/5<br>Con: 12/4	Int1: 65 ± 8<br>Int2: 65 ± 11<br>Con: 66 ± 8	FEV1/FVC < 70%	Int1: Land-based Liu Zi Jue<br>Int2: Water-based Liuzijue	60 min	2	12	120	Supervised	6MWD
Mehri et al., 2007	Int: 20<br>Con: 18	NR	NR	mMRC dyspnea score ≥ 3, barthel index	Treadmill	NR	2	8	NR	Supervised	VO_2max

				< 50							
Mirza et al., 2020	Int: 20<br>Con: 18	Int: 20/0<br>Con: 17/1	Int: 62.3 ± 6.6<br>Con: 65.5 ± 8.2	NR	Combined endurance and strength training	30 min	7	1	420	Supervised	2MWD
Moy et al., 2021	Int: 36<br>Con: 37	Int: 17/19<br>Con: 27/8	Int: 69.6 ± 7.5<br>Con: 70.5 ± 9.2	NR	Aerobic training	60 min	1-2	24	60-120	Supervised	6MWD
Na et al., 2023	Int: 20<br>Con: 20	Int: 103/11<br>Con: 99/15	Int: 70.242 ± 8.3980<br>Con: 69.183 ± 9.0873	NR	Aerobic training	60 min	7	8	420	Unsupervised	6MWD
Neves et al., 2018	Int: 10<br>Con: 10	Int: 6/4<br>Con: 6/4	Int: 63.8 ± 8.1<br>Con: 63.5 ± 7.8	NR	Whole-body vibration training	8 min	3	12	24	Supervised	6MWD
Niu et al., 2014	Int: 20<br>Con: 19	Int: 19/1<br>Con: 18/2	Int: 59.7 ± 2.76<br>Con: 61.3 ± 2.89	NR	Tai Chi	50 min	7	24	350	Mixed	6MWD
Pleguezuelos et al., 2013	Int: 26<br>Con: 25	Int: 26/0<br>Con: 25/0	Int: 68.4 ± 8.9<br>Con: 71.3 ± 8.0	NR	Whole-body vibration training	40 min	3	6	120	Supervised	6MWD
Pradella et al., 2015	Int: 29<br>Con: 15	Int: 23/6<br>Con: 13/2	Int: 62.4 ± 10.7<br>Con: 65.3 ± 8	NR	Home exercise	NR	3	NR	NR	Unsupervised	6MWD
Ranjita et al., 2016	Int: 36<br>Con: 36	Int: 36/0<br>Con: 36/0	Int: 53.69 ± 5.66<br>Con: 54.41 ± 5.40	FEV1 < 65%, FEV1/FVC < 70%	Yoga	90 min	6	12	540	Supervised	6MWD
Santiworakul et al.,	Int: 10<br>Con: 10	Int: 10/0<br>Con: 10/0	Int: 70.80 ± 2.66<br>Con: 68.30 ± 4.57	FEV1/FVC < 70%, FEV1 <	Strength training	NR	9	8	NR	Unsupervised	6MWD

2011				50%							
Takahashi et al., 2011	Int: 35<br>Con: 32	Int: 35/0<br>Con: 32/0	Int: 72.5 ± 7.04<br>Con: 72.4 ± 7.01	FEV1 < 80%	Low-intensity seated gymnastics	NR	3	12	NR	Unsupervised	6MWD
Wu et al., 2018	Int1: 16<br>Int2: 17<br>Con: 17	Int1: 14/2<br>Int2: 13/4<br>Con: 14/3	Int1: 67 ± 8<br>Int2: 64 ± 8<br>Con: 66 ± 9	FEV1/FVC < 70%, FEV1 < 80%	Int1: Liu Zi Jue<br>Int2: Liu Zi Jue combined with resistance band training	Int1: 32-35 min<br>Int2: 60 min	6	24	Int1: 180<br>Int2: 360	Mixed	6MWD
Zhu et al., 2018	Int: 30<br>Con: 30	Int: 28/2<br>Con: 29/1	Int: 67.87 ± 5.22<br>Con: 68.10 ± 6.57	FEV1/FVC < 70%, FEV1 < 80%	Tai Chi	40-50 min	3	12	120-150	Supervised	6MWD
Zhu et al., 2025	Int: 27<br>Con: 27	Int: 18/9<br>Con: 18/9	Int: 65.3 ± 3.9<br>Con: 64.6 ± 3.9	NR	Mawangdui Daoyin Qigong	60 min	5	12	300	Unsupervised	6MWD

Abbreviations: Int, intervention groups; Con, control groups; M, male; F, female; HIIT, high intensity interval training; NR, no report; 6MWD, 6-minute walk distance; VO_2max, maximal oxygen uptake; ESW, endurance shuttle walk; mMRC: modified medical research council; FVC: Forced vital capacity; FEV1, forced expiratory volume in one second.

Table S3. Results of meta-regression.

Modalities	_ES	Coef.	Std. Err.	t	p > |t|	95% CI
**Session duration**	subgroup	0.2090847	0.2728897	0.77	0.450	-0.3482304, 0.7663997
	_cons	39.20588	15.4023	2.55	0.016	7.75019, 70.66156
**Frequency**	subgroup	-4.848282	1.549963	-3.13	0.003	-7.991752, -1.704813
	_cons	74.03169	9.551718	7.75	<0.001	54.65991, 93.40347
**Weekly time**	subgroup	-0.0580177	0.0506836	-1.14	0.262	-0.1616774, 0.045642
	_cons	63.5248	13.39026	4.74	<0.001	36.13864, 90.91096
**Type of intervention**	subgroup	-0.1822679	14.37115	-0.01	0.990	-29.42059, 29.05606
	_cons	49.24377	19.4232	2.54	0.016	9.726967, 88.76058
**Supervision status**	subgroup	-15.77907	13.91524	-1.13	0.266	-44.19779, 12.63965
	_cons	70.15543	19.93446	3.52	0.001	29.44383, 110.867

Abbreviations: Coef., coefficient; Std. Err., standard error; t, t-test statistic; p, probability; CI, confidence interval; 6MWD, 6-minute walk distance.

Table S4. Results of Egger's test

**Std_EFF**	**Coef.**	**Std. Err.**	**t**	**p > |t|**	**95% CI**
Slope	34.69244	6.687819	5.19	0.000	21.12892, 48.25597
Bias	0.7255953	0.5003472	1.45	0.156	-0.2891558, 1.740346

Abbreviations: Coef, coefficient; Std. Err, standard error; t, t-test statistic; p, probability; CI, confidence interval.

Table S5. GRADE summary of evidence.

Certainty assessment							No. of participants		Effect		Certainty	Importance
No. of studies	Study design	Risk of bias	Inconsistency	Indirectness	Imprecision	Other considerations	Experimental	Control	Relative (95% CI)	Absolute		
34	RCT	Serious1	Serious2	No serious indirectness	No serious imprecision	None	1124	1089	-	WMD 46.66 higher (35.94 to 57.38 higher)	⊕⊕○○ Low	-

Note: RCT: randomized controlled trial, WMD: weighted mean difference, CI: confidence interval.

[1]Performance bias

[2]Substantial heterogeneity

GRADE Working Group grades of evidence: ⊕⊕⊕⊕, High certainty: we are very confident that the true effect lies close to that of the estimate of the effect; ⊕⊕⊕○, Moderate certainty: we are moderately confident in the effect estimate: the true effect is likely to be close to the estimate of the effect, but there is a possibility that it is substantially different; ⊕⊕○○, Low certainty: our confidence in the effect estimate is limited: the true effect may be substantially different from the estimate of the effect; ⊕○○○, Very low certainty: we have very little confidence in the effect estimate: the true effect is likely to be substantially different from the estimate of effect.
